# Cognitive dysfunction in MASLD is linked to platelet activation

**DOI:** 10.3389/fnins.2026.1909908

**Published:** 2026-09-01

**Authors:** Jiaqin Chen, Chi Cheng, Haiying Wang, Tian Yu

**Affiliations:** 1 Department of Anesthesiology, Affiliated Hospital of Zunyi Medical University, Zunyi, China; 2 Guizhou Key Laboratory of Anesthesia and Organ Protection, Zunyi Medical University, Zunyi, China

**Keywords:** central nervous system, cognitive dysfunction, fatty liver disease, metabolic dysfunction-associated steatotic liver disease, platelet activation

## Abstract

Metabolic dysfunction-Associated Steatotic liver disease (MASLD) is one of the most common systemic diseases causing chronic liver diseases. Current evidence indicates that cognitive-related deficits occur frequently in MASLD, causing significant impacts on patients and their families, and even possibly increasing the economic and psychological burdens on healthcare, productivity, and nursing staff. However, there are currently no effective treatment methods. This article reviews the pathogenesis of cognitive dysfunction in MASLD, finding that platelet activation is elevated. Furthermore, recent studies have found that platelets can send and receive signals to the central nervous system (CNS), serving as an important bridge between the brain and peripheral organs. This article reviews changes in platelets associated with MASLD and investigates the role of platelet activation, with the goal of offering novel approaches for diagnosing and treating cognitive dysfunction in MASLD.

## 1 Introduction

MASLD is a systemic disorder characterized not only by hepatic manifestations—including nonalcoholic steatohepatitis (NASH), liver fibrosis, cirrhosis, and hepatocellular carcinoma-but also by extrahepatic complications such as cardiovascular disease and cognitive impairment (Yu et al., 2022; Wang et al., 2025; Vataja et al., 2025). Recent studies have established a link between MASLD and cognitive impairment, particlarly in the domains of attention and memory, which substantially reduces patients’ quality of life. Studies have found that platelets play a significant role in MASLD progression, and in this condition, platelets are activated (Ye and Xu, 2026). Evidence suggests that platelets may play an important role in connecting peripheral metabolic and inflammatory pathways to the CNS (Narayanan, 2024). Their cargo of neurotransmitters enables them to modulate neuronal and blood–brain barrier function, suggesting a role in transmitting pathological signals via the liver-brain axis (Malehmir et al., 2019). Furthermore, clinical trials hint that antiplatelet therapy could slow liver disease progression (Li S. Y. et al., 2022; Gautam et al., 2023). However, the underlying mechanisms mediating this association remain largely unknown. In particular, the dual role of platelets—as both a peripheral inflammatory mediator and a potential bridge linking hepatic pathology to central nervous system dysfunction—remains to be fully elucidated. Therefore, targeting platelet function offers a dual prospect: as a potential diagnostic indicator for early identification of cognitive risk, and as a novel therapeutic strategy for MASLD-associated cognitive decline-an approach that, to our knowledge, has not been systematically investigated.

## 2 Underlying mechanisms: cognitive impairment in MASLD

**Figure 1:**
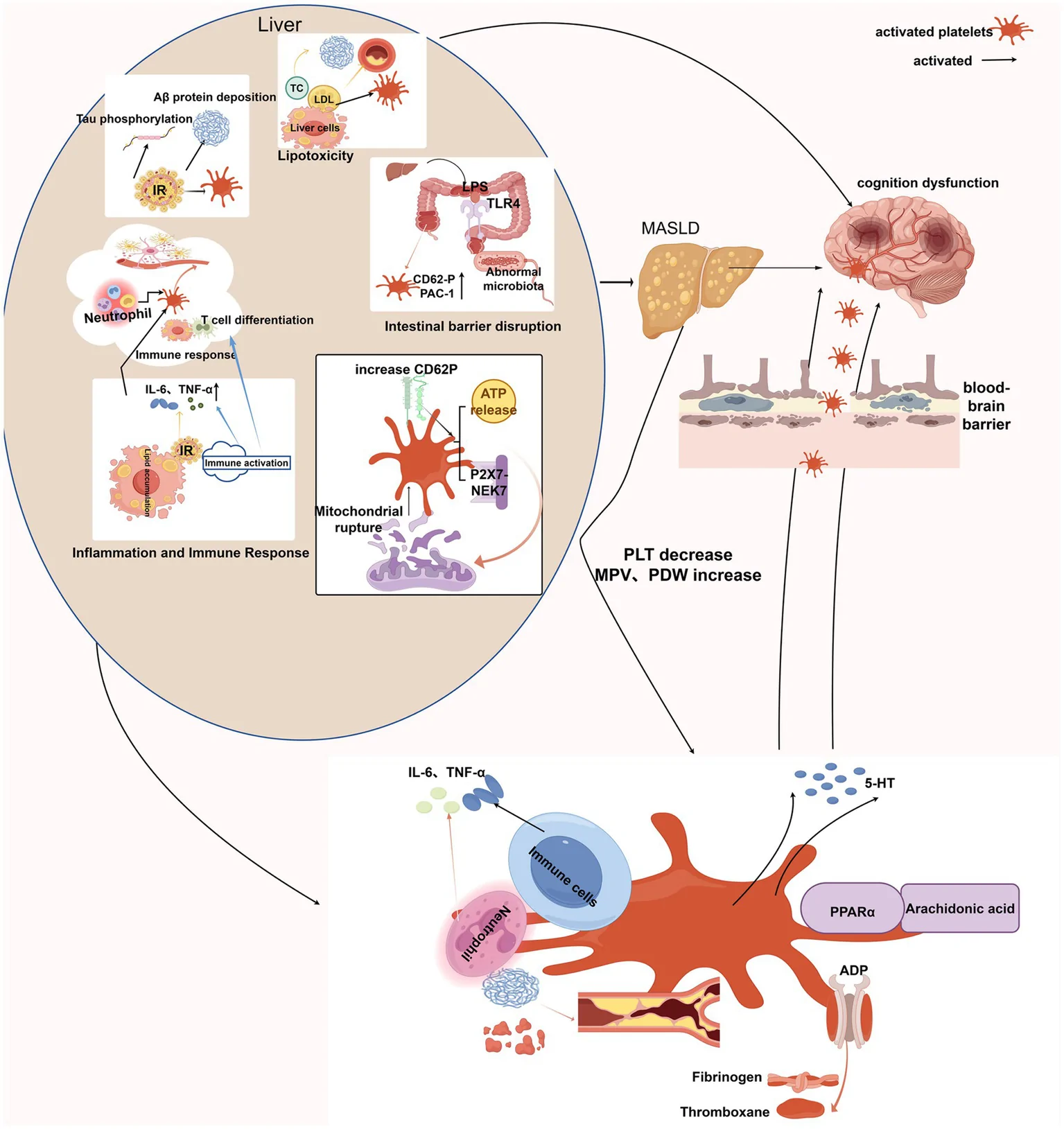
Mechanism of platelets participating in cognition dysfunction in MASLD. Insulin resistance, lipotoxicity, inflammation and immune response, intestinal barrier damage, mitochondrial dysfunction and cerebrovascular disease lead to platelet activation. The activated platelets damage the blood–brain barrier through multiple ways, resulting in cognitive dysfunction in MASLD. Created with Figdraw 2.0.

MASLD, currently the most common cause of chronic liver disease, is frequently associated with cognitive dysfunction (Le et al., 2025). Cross-sectional studies have reported that the prevalence of cognitive impairment in NAFLD (the former term for MASLD) ranges from 12 to 41%, depending on the assessment method used (Parikh et al., 2024). Recent literature indicates that MASLD is negatively correlated with cognitive function and cerebral integrity, and increases the risk of cerebrovascular lesions (He et al., 2025). Although the growing body of epidemiological and imaging evidence suggests a connection between the brain and the liver, it does not reveal the underlying causal pathways. Hepatic lipid accumulation, insulin resistance, inflammation, immune activation, intestinal barrier disruption, mitochondrial dysfunction, and vascular lesions may all represent common pathways linking the liver and the brain. Platelets, as key sensors and amplifiers of systemic inflammation, are increasingly recognized as potential mediators of the liver–brain axis. However, direct evidence for this link—particularly regarding MASLD-associated cognitive decline—remains scarce, underscoring the need for further investigation (Figure 1).

### 2.1 Lipid toxicity mediates cognitive impairment in MASLD

Dyslipidemia is a hallmark of MASLD, primarily characterized by elevated levels of triglycerides (TG) and low-density lipoprotein cholesterol (LDL-C) (Syed-Abdul, 2023; Rao et al., 2023). When hepatic *de novo* lipogenesis increases and mitochondrial *β*-oxidation is insufficient, the influx of free fatty acids (FFA) rises, and excessive FFA intake further overwhelms the very-low-density lipoprotein (VLDL) transport capacity, leading to lipid accumulation, fatty degeneration of hepatocytes, and ultimately elevated TG levels (Chen et al., 2025). In MASLD, activation of sterol regulatory element-binding protein 2 (SREBP-2) promotes cholesterol synthesis and increases VLDL secretion, providing LDL precursors, while downregulation of hepatic LDL receptor impairs LDL clearance, collectively resulting in elevated LDL-C (Syed-Abdul, 2023). MASLD-associated lipotoxicity impairs cognitive function through a self-reinforcing cascade that begins in the cerebral vasculature and culminates in platelet hyperactivation. Elevated triglycerides and LDL cholesterol initiate vascular damage by promoting amyloid-*β* (Aβ) deposition, atherosclerosis, and endothelial dysfunction, with oxidized LDL (ox-LDL) further exacerbating blood–brain barrier disruption and neuroinflammation (Li H. et al., 2025; Wen et al., 2024; Farias et al., 2025; Yang et al., 2017). This compromised vascular milieu not only directly injures neural tissue but also sets the stage for platelet-mediated thrombosis. Indeed, both LDL and ox-LDL directly prime platelets for activation, adhesion, and aggregation via the arachidonic acid-TXA2 pathway and enhanced procoagulant activity (Kelem et al., 2023; Pedreno et al., 2001). Moreover, the broader lipotoxic environment—independent of specific lipoprotein fractions—potentiates platelet responsiveness by increasing fibrinogen binding, upregulating activation marker P-selectin (CD62-P), and activating PPARα-dependent signaling cascades (Ye and Xu, 2026; van Geffen et al., 2020). Thus, via this integrated vascular-platelet axis, lipotoxicity establishes a vicious cycle wherein endothelial injury and platelet hyperreactivity mutually reinforce one another, ultimately driving cerebral hypoperfusion, neurovascular unit dysfunction, and cognitive decline (Li L. et al., 2022).

### 2.2 Insulin resistance (IR) mediated cognitive impairment in MASLD

IR is a key pathophysiological mechanism in MASLD and a significant risk factor for neurodegeneration and cognitive dysfunction. Hepatic lipid overload promotes the generation of lipotoxic metabolites—including diacylglycerol and ceramides—that directly impair IRS/PI3K/AKT signaling, thereby inducing IR (Truong and Lee, 2025; Gastaldelli et al., 2026). IR drives cognitive impairment through a bifurcated pathogenic axis, encompassing both direct central neurotoxicity and peripheral platelet-mediated cerebrovascular compromise. On the central front, IR disrupts neuronal insulin signaling via reduced receptor expression and impaired IRS1/PI3K pathway, leading to increased oxidative stress and mitochondrial dysfunction, which directly inflict neuronal damage and synaptic impairment (Yavari et al., 2025; Nuzzo et al., 2015; Gutierrez-Tordera et al., 2025; Ahlawat et al., 2025). This central insulin signaling defect further exacerbates microtubule-associated protein (tau) hyperphosphorylation, neuroinflammation, and blood–brain barrier (BBB) compromise, collectively fostering a neurotoxic milieu (Sedzikowska and Szablewski, 2021; Malin et al., 2022). Concurrently, IR-induced hypometabolism reduces cerebral glucose utilization and perfusion, which not only impairs Aβ clearance but also promotes its accumulation and deposition (Nakhaee et al., 2024). Compounding these direct cerebral effects, IR exerts profound systemic influences on platelet function. Hyperglycemia and hyperinsulinemia prime platelets for heightened reactivity (Arnoldussen and Witkamp, 2021), enhancing aggregation, fibrinogen binding, and TXA2 production, which promote microthrombus formation and compromise cerebral blood flow (Dalbeni et al., 2022). Moreover, in the IR state, platelets become hyperresponsive to subthreshold stimuli, accelerating their consumption and further amplifying thrombotic risk (Gonzalez-Mendoza et al., 2025). Ultimately, the neuroinflammation further amplifies platelet reactivity, while platelet thrombi exacerbate cerebral hypoxia, together accelerating the trajectory toward cognitive impairment.

### 2.3 Inflammatory pathways leading to cognitive dysfunction in MASLD

A persistent low-grade inflammatory state permeates the entire pathological course of MASLD. Excessive hepatic lipid accumulation and IR can significantly increase the release of pro-inflammatory cytokines, including tumor necrosis factor-alpha (TNF-*α*), interleukin-6 (IL-6), and others, thereby amplifying systemic inflammation and driving the progression of MASLD and its related complications (Zisis et al., 2025; Li et al., 2024b). Furthermore, the increased release of pro-inflammatory cytokines has been closely associated with cognitive dysfunction, suggesting that systemic inflammation may serve as a key mechanistic link between MASLD and neurocognitive decline. Pro-inflammatory cytokines TNF-α, IL-6 released from the fatty liver cross the BBB to activate neuroinflammation and synaptic impairment (Gu et al., 2025). This direct pathway facilitates the fusion of cytokines with neuronal lipid rafts or platelet-derived exosomes, enabling their transport into the brain parenchyma, where they amplify neuroinflammatory signals and activate astrocytes and microglia (Boncler et al., 2024; Huang et al., 2021). Simultaneously, inflammatory mediatorscompromise cerebral perfusion by increasing blood viscosity, erythrocyte aggregation, and platelet reactivity. These neuroinflammatory and hemodynamic disturbances establish a synergistic cycle—neuroinflammation impairs vascular function, while hypoperfusion reduces clearance of inflammatory mediators—culminating in progressive cognitive decline (Li et al., 2024a).

### 2.4 Involvement of immune cells in MASLD cognitive impairment

Sustained activation of immune signaling pathways promotes the progression of MASLD. Furthermore, metabolic dysregulation has been shown to drive immune cell polarization, whereby polarized immune cells assault the CNS through both direct and indirect pathways, collectively accelerating cognitive decline in MASLD (Schwarzler et al., 2024). On the direct front, pro-inflammatory immune cells infiltrate adipose tissue, secreting a wide array of inflammatory mediators that sustain systemic inflammation (Wang et al., 2025; Frasca and Blomberg, 2020). This systemic inflammatory milieu subsequently facilitates the recruitment of these activated immune cells across the BBB into the brain parenchyma, where they directly induce astrocyte death and neuronal dysfunction, thereby igniting neuroinflammation and cognitive impairment (Coulibaly, 2022). Emerging evidence highlights that platelets play a critical role in the innate immune system (Mandel et al., 2022). Under inflammatory conditions, activated platelets adhere to and interact with circulating immune cells-most notably (Hally et al., 2020) promoting neutrophil activation and recruitment, which elevates IL-6 levels and exacerbates cognitive dysfunction (Coulibaly, 2022). Furthermore, platelet-derived mediators recruit T cells to sites of inflammation and exacerbate T-cell-dependent cerebrovascular and BBB injury, thereby impairing neurocognitive function (Koupenova et al., 2022). Critically, these direct immune-mediated neuronal injury and indirect platelet-immune crosstalk do not operate in isolation; rather, they establish a synergistic feedback loop wherein platelet activation enhances immune cell infiltration, while immune-derived cytokines further prime platelet reactivity, collectively amplifying the neuroinflammatory burden and hastening cognitive decline in MASLD.

### 2.5 Intestinal barrier disruption mediates cognitive impairment in MASLD

During the development of MASLD, levels of various microbial products and host pattern recognition receptors (PRRs) are elevated. Of these, the most extensively studied are lipopolysaccharide (LPS) and Toll-like receptor 4 (TLR4), whose upregulation is indicative of increased intestinal permeability and microbial translocation (Yang et al., 2023). During MASLD progression, gut dysbiosis accompanies elevated bile acids (BA). The gut microbiota comprises predominantly Bacteroidetes, Firmicutes, Actinobacteria, Proteobacteria, and Verrucomicrobiota, and is involved in nutrient metabolism, immune system maturation, maintenance of the intestinal epithelial barrier, and pathogen resistance (Zhu et al., 2024). In MASLD, this delicate equilibrium is disrupted, manifesting as increased abundance of Firmicutes, Actinobacteria, and Proteobacteria, alongside a significant reduction in Bacteroidetes—a signature of pronounced dysbiosis (Garcia-Mateo et al., 2024; Qin et al., 2025). The altered Firmicutes/Bacteroidetes ratio disrupts the intestinal barrier by compromising tight junction integrity and degrading the mucus layer, thereby increasing permeability (Suppli et al., 2021; Kirundi et al., 2023). Concurrently, specific bacterial taxa exert distinct pathogenic effects. For instance, elevated Proteobacteria, particularly Enterobacteriaceae, serve as a reservoir of LPS-endotoxin, directly fueling in intestinal permeability leads to inflammation (Soto et al., 2024). Conversely, among Firmicutes, Romboutsia and Turicibacter exhibit increased abundance (Yang et al., 2026). This alteration disrupts energy provision for colonic epithelial cells and attenuates anti-inflammatory signaling essential for epithelial homeostasis. Adding to this structural compromise, the downregulation of interleukin-22 (IL-22)—a protective antimicrobial cytokine secreted by small intestinal lymphocytes—impairs epithelial cell differentiation and maturation, promoting excessive absorption of lipids and carbohydrates and further weakening barrier function (Zhang et al., 2024). Compounding these microbial-driven defects, elevated bile acids in MASLD damage cellular membranes, stimulate ROS production, and interfere with Farnesoid X receptor/Takeda G-protein-coupled receptor 5(FXR/TGR5)-mediated protective signaling, collectively promoting tight junction disassembly and perpetuating increased permeability (Zhou et al., 2025; Ji et al., 2020; Yu et al., 2025). This leaky gut permits translocation of microbial products—most notably LPS—into the portal circulation, where they activate hepatic TLR-4/NF-κB signaling and drive pro-inflammatory cytokine release (Shi et al., 2023). These inflammatory mediators, in turn, further compromise intestinal barrier integrity, establishing a self-reinforcing loop that sustains systemic inflammation. Through the microbiota–gut–brain axis, this systemic inflammatory burden reaches the CNS, triggering neuroinflammation and contributing to cognitive decline (Wu et al., 2021). Notably, the gut microbiome also exerts distal effects via platelet modulation: dysbiosis-associated microbial metabolites entering the plasma induce platelet hyperactivation and degranulation, as evidenced by positive correlations between Firmicutes abundance and platelet activation markers (CD62P, PAC-1) (Liu et al., 2020). This platelet hyperactivation releases 5-HT and amplifies pro-inflammatory cytokine secretion, further disrupting BBB integrity and driving neurocognitive dysfunction (Rust et al., 2023). Thus, the gut microbiota impacts brain health through both direct inflammatory pathways and indirect platelet-mediated mechanisms, which converge in a synergistic axis linking intestinal dysbiosis to cognitive impairment in MASLD (Rust et al., 2023).

### 2.6 Mitochondrial dysfunction involves cognitive impairment in MASLD

Mitochondrial dysfunction is a key pathological feature of MASLD and bridges MASLD to cognitive decline via a platelet-dependent cascade (Li X. et al., 2025). In the early stages of MASLD, mitochondria transiently redistribute to perinuclear lipid droplets as a compensatory mechanism to buffer fatty acid overload and mitigate lipotoxicity. With disease progression, however, this adaptive sequestration collapses, unleashing lipotoxic stress that drives overt mitochondrial dysfunction (Sun et al., 2024). Given that neurons are exquisitely dependent on mitochondrial integrity due to their immense metabolic demands, even mild bioenergetic deficits can destabilize synapses and precipitate cognitive impairment. This neuronal vulnerability is exacerbated by a vicious cycle in which mitochondrial dysfunction drives cellular and brain aging via impaired energy generation, excessive ROS production, disrupted dynamics, and defective mitophagy—abnormalities that reciprocally interact with Aβ and tau pathology to sustain neuroinflammation and progressive neurodegeneration (D'Alessandro et al., 2025). Mitochondrial–platelet crosstalk in MASLD operates as a reciprocal amplification loop: mitochondrial dysfunction activates platelets via P-selectin upregulation, and activated platelets, in turn, release ATP that promotes DRP1-mediated mitochondrial fragmentation in hepatocytes via purinergic receptor P2X7-NIMA-related kinase 7 (P2X7-NEK7) signaling (Michailidou et al., 2023; Cao et al., 2026). This feeds back to exacerbate mitochondrial injury, while platelet-derived mitochondria accumulating at synapses drive oxidative stress, energy failure, and synaptic dysfunction—ultimately accelerating cognitive decline (Bhat et al., 2024). Thus, the bidirectional mitochondrial–platelet axis propagates pathology from liver to brain and represents a potential therapeutic target.

### 2.7 Alzheimer’s disease (AD) and MASLD-related cognitive impairment

Platelet hyperactivation plays a pivotal role in shaping the neurovascular and inflammatory environment of AD, linking metabolic liver disease to Alzheimer’s neurodegeneration (Weinstein et al., 2022). AD and MASLD share overlapping mechanisms as evidenced by common phenotypes (reduced brain volume, impaired perfusion, and platelet hyperreactivity) ms (Mai and Mao, 2023) and AD (Falcon et al., 2024; Zhou et al., 2024; Ge, 2024). Chronically hyperactivated platelets in MASLD contribute to AD-like pathology by elevating Aβ burden, promoting its aggregation, and forming microthrombi that reduce cerebral perfusion (Moretti et al., 2022; Yilmaz et al., 2023; Palix et al., 2022; Xiong et al., 2025; Yang et al., 2023; Ramos-Cejudo et al., 2022; Koch-Paszkowski et al., 2025). Moreover, activated platelets release matrix metalloproteinases (MMPs), which degrade fibrous cap collagen and precipitate atherosclerotic plaque destabilization. Plaque rupture exposes highly thrombogenic substrates, triggering massive platelet aggregation, occlusive cerebral thrombosis, and subsequent cognitive decline (Jebari-Benslaiman et al., 2022). Therefore, the MASLD-AD connection is not unifactorial but emerges from synergistic crosstalk between vascular components and platelets, which collectively link metabolic-associated liver disease to AD’s pathology.

In summary, MASLD-related cognitive impairment is likely to be mediated by multiple interacting mechanisms, including insulin resistance, lipotoxicity, chronic inflammation, immune dysregulation, mitochondrial dysfunction and intestinal barrier disruption. Platelet activation plays a pivotal role in each of these pathways, exacerbating brain injury through the promotion of thrombosis, neuroinflammation, and Aβ deposition. Thus, platelet activation serves not only as a convergence point for diverse pathogenic signals underlying MASLD-associated cognitive decline but also as a functional bridge linking peripheral organ damage to central neurodegenerative changes.

## 3 Platelet changes in cognitive dysfunction of MASLD

Notably, platelets have emerged as candidate biomarkers for assessing liver fibrosis and the progression of liver failure (Michalak et al., 2021). MASLD drives a multifaceted platelet alteration, marked not only by thrombocytopenia but also by an activated morphological profile—including elevated Mean Platelet Volume (MPV) and Platelet Distribution Width (PDW)—and heightened granule secretion, collectively indicating a state of enhanced platelet reactivity. Given their broad phenotypic and functional remodeling during MASLD progression, platelets may serve as mechanistic intermediaries linking hepatic pathology to extrahepatic complications, including cognitive dysfunction.

### 3.1 Decreased platelet count in MASLD

The liver serves as the primary regulatory organ for platelets, promoting their production through the secretion of thrombopoietin (TPO). MASLD presents a paradoxical platelet phenotype, characterized by a quantitative reduction in platelet count coexisting with a state of qualitative hyperactivation. This thrombocytopenia arises from a dual-hit mechanism: persistent inflammation and fibrosis. Fibrosis develops secondary to impaired platelet production caused by diminished hepatic TPO synthesis, and enhanced peripheral consumption mediated by interleukin-1(IL-1)-driven von Willebrand factor (vWF) release from activated endothelium (Nanizawa et al., 2024; Reiche et al., 2024). Paradoxically, although reduced platelet counts are epidemiologically linked to MASLD progression, accumulating evidence indicates that this numerical decline does not equate to functional silencing. Instead, the residual platelets in advanced liver disease exhibit heightened activation, as supported by elevated urinary levels of 11-dehydrothromboxane B2 (11-dh-TXB2), a stable marker of *in vivo* platelet activity. Moreover, the very the very clearance mechanism responsible for platelet elimination-via interactions between (Castelli et al., 2025). surface receptors on target cells- may paradoxically precipitate this hyperactive state (Minciuna et al., 2024). Ultimately, these hyperresponsive platelets are posited to disrupt brain homeostasis by damaging the blood–brain barrier, inducing cerebral microthrombi, and releasing neurotoxic substances, thereby linking hepatic inflammation to cerebral dysfunction.

### 3.2 Elevated MPV in MASLD

Patients with MASLD typically exhibit an elevated MPV. Two potential mechanisms may account for this phenomenon. The first involves a pro-inflammatory drive: upregulated IL-1, IL-6, and TNF-*α* stimulate hepatic thrombopoietin synthesis, The first involves a pro-inflammatory drive: IL-1, IL-6, and TNF-αstimulate hepatic thrombopoietin synthesis, which promotes megakaryocyte ploidy and cytoplasmic expansion thereby generating larger andmore reactive platelets (Korniluk et al., 2019). The second mechanism operates through a. vascular anti-aggregatory deficit insulin resistance suppresses endothelial production of prostacyclin and nitric oxide, removing critical vasodilatory and platelet-inhibitory signals, which further sensitizes platelets and contributes to MPV elevation (Varol et al., 2010). The combined effect—oversized platelets operating in a permissive vascular environment—establishes a hyperreactive phenotype that links MASLD to thrombotic and cognitive risks.

### 3.3 Increased platelet distribution width (PDW) in MASLD

Patients with MASLD typically present with an elevated PDW compared to healthy controls (Milovanovic Alempijevic et al., 2017). The chronic inflammatory milieu characteristic of MASLD releases a spectrum of inflammatory mediators, driving platelet activation and subsequent morphological and structural remodeling—ultimately culminating in increased PDW (Ferencova et al., 2023). Concurrently, heightened platelet consumption in MASLD accelerates the release of larger, newly generated platelets from the bone marrow as a compensatory response, further contributing to PDW elevation (Chen et al., 2022; Zhang and Wang, 2025). These two processes are not independent but functionally intertwined: inflammation-driven platelet activation not only remodels existing platelets but also accelerates their clearance, thereby amplifying the demand for megakaryocyte output and perpetuating the release of immature, oversized platelets. This reflects systemic platelet hyperreactivity underlying the vascular risks associated with MASLD.

### 3.4 Increased release of platelet granules in MASLD

Platelet-activating factor (PAF) has been implicated in the progression of MASLD. PAF binds to PAFR on the platelet membrane to directly trigger platelet activation. Activated platelets subsequently release bioactive mediators including 5-HT and CD40L, thereby driving disease progression. First, pathological conditions- inflammation, IR, and oxidative stress-induce platelets to generate PAF,which upon receptor binding triggers platelet activation and promotes 5-hydroxytryptamine (5-HT) release. Notably, elevated 5-HT levels correlate positively with the non-alcoholic fatty liver disease activity score (NAS), establishing a clinically relevant autocrine loop that perpetuates platelet hyperreactivity (Yin et al., 2022). Second, activated platelets surface-translocate and secrete CD40 ligand (CD40L), from *α*-granules,a molecule that bridge platelet activation with adaptive immunity and vascular inflammation, thereby amplifying the systemic inflammatory milieu beyond the platelet compartment (Ma et al., 2022). Third, platelet-derived C-X-C motif chemokine ligand 5 (CXCL5), directly enhance hepatocyte lipotoxicity in high-fat diet models (Kushiro et al., 2025) Together, these pathways position platelets as a pathogenic hub that amplify their own activation and injure hepatocytes.

Collectively, studies have shown that platelet activation markers (such as MPV and PDW) are elevated in MASLD. Several platelet-related indices have emerged as promising predictors of disease severity, such as the platelet-to-hepatocyte ratio (PHR), gamma-glutamyl transpeptidase-to-platelet ratio (GPR), Fibrosis-4 index (FIB-4), and systemic immune-inflammation index (SII). These observations underscore the pivotal role of platelets in MASLD progression. Nevertheless, the precise mechanisms driving platelet activation in this context remain incompletely understood and warrant further investigation.

## 4 Platelet activation in neurodegenerative diseases

Platelet activation is the process in which resting platelets are stimulated by physiological or pathological factors. This may lead to platelet activation-a process involving a cascade of biochemical, structural, and morphological changes, notably the development of pseudopodia (Liao et al., 2025; Peng et al., 2024). This process is primarily mediated by G-protein-coupled receptors (GPCRs) and immunoreceptor tyrosine-based activation motifs (ITAMs). Upon receptor engagement, downstream signaling cascades are initiated, facilitating integrin activation and granule exocytosis, which collectively drive platelet aggregation and further amplify activation (Nieman and Neeves, 2023). Through mechanisms such as thrombus formation, release of pro-inflammatory cytokines, and dynamic crosstalk with various cell types, platelet activation contributes to the pathogenesis of multiple diseases (Tian et al., 2025). Studies have also shown that platelet activation biomarkers, such as the glycoprotein (IIb–IIIa αIIb*β*3) complex and P-selectin, can serve as potential predictive biomarkers of cognitive decline in patients with neurodegenerative diseases (Rodriguez Moore et al., 2024).

### 4.1 Platelet activation and AD

AD is the most common neurodegenerative disorder. Its core pathological features include the deposition of β-amyloid (Aβ), hyperphosphorylation of tau, chronic neuroinflammation, and neuronal loss (Song et al., 2024). The evidence indicates that the structural integrity of vulnerable brain regions in AD patients is associated with platelet activation (Palix et al., 2022). Upon activation, platelets secrete platelet-derived growth factor (PDGF-BB) to induce cerebral pericyte dysfunction. This cascade reaction is supported by a cohort study in Molecular Neurodegeneration showing elevated cerebrospinal fluid (CSF) platelet-derived growth factor receptor-*β*(PDGFRβ) levels in cognitively impaired patients and an association between decreased plasma sPDGFRβ and BBB damage in Alzheimer’s (Li et al., 2026) This observation implies that persistent platelet activation and excess PDGF-BB may trigger progressive pericyte loss, disrupt cerebrovascular homeostasis, and aggravate AD neuropathology. In AD patients, platelets serve as key peripheral reservoirs of Aβ (Rodriguez Moore et al., 2024). These cells accelerate AD-related neurodegeneration via Aβ-induced platelet activation, cerebral microthrombosis, and tau-mediated synaptic injury, collectively constituting a self-amplifying pathogenic loop (Rodriguez Moore et al., 2024; Slachevsky et al., 2017). These processes, centered on platelet activation, drive Aβ pathology, thrombus formation, and tau aggregation, thereby accelerating AD neurodegeneration.

### 4.2 Platelet activation and PD

PD is characterized by core pathological changes including apoptosis of dopaminergic neurons, abnormal *α*-synuclein aggregation, Lewy body formation, and chronic neuroinflammation (Musaro et al., 2026). In patients with PD, comparable platelet activation processes – including adhesion, aggregation, and degranulation – were observed (Beura et al., 2022). First, activated platelets release high levels of *α*-synuclein, which can induce misfolding and aggregation of α-synuclein in brain neurons, thereby promoting Lewy body formation-a process supported by the observation of comparable platelet activation (adhesion, aggregation, degranulation) in PD patients (Gautam et al., 2023; Maszka et al., 2023). Second, platelet-derived ROS and inflammatory factors exacerbate oxidative stress and apoptosis in dopaminergic neurons (Takahashi and Takahashi, 2019). Third, at the mitochondrial level, platelets from patients with PD exhibit decreased complex I activity-a defect that mirrors the well established mitochondrial dysfunction in the substantia nigra-and impairment induces excessive ROS, further accelerating neuronal loss (Beura et al., 2022). Finally, beyond these structural and metabolic abnormalities, platelets harbor synaptic pathology: their dense granules store dopamine and its transporter; however, activated platelets exhibit decreased expression of vesicular monoamine transporter 2 mRNA expression, which impairs vesicular dopamine packaging and may contribute to the synaptic failure in PD (Gautam et al., 2023; Beura et al., 2022). Together, these processes establish platelets as a peripheral readout of central neurodegeneration and active contributors to disease progression.

### 4.3 Platelet activation and multiple sclerosis (MS)

MS is an autoimmune neurodegenerative disease characterized by CNS inflammatory demyelination, blood–brain barrier disruption, and neuronal damage (Matias-Guiu et al., 2017). In MS, platelets show an abnormal activation state (Langer et al., 2012). The activation of platelets actively participates in the pathological process of MS by simultaneously attacking the myelin sheath of the CNS and the vascular barrier, thereby jointly exacerbating neuroinflammation and tissue damage. First, platelet activation induces the transition of macrophages and other immune cells from a resting to a reactive state, promoting excessive myelin damage and resulting in axonal demyelination and tissue injury (Ferrer-Raventos and Beyer, 2021). Second, activated platelets release large amounts of substances, including MMPs. MMPs degrade the vascular basement membrane, thereby increasing blood–brain barrier permeability. This allows peripheral immune cells and toxic substances to invade the CNS parenchyma and initiate local demyelinating lesions (Cheng et al., 2024). Therefore, in addition to their typical role in hemostasis, platelets exert immunomodulatory and vascular-damaging effects in MS. Their activation can exacerbate neuroinflammation and tissue damage associated with MS.

### 4.4 Platelet activation and amyotrophic lateral sclerosis (ALS)

ALS is a fatal neurodegenerative disease of motor neurons, characterized by motor neuron apoptosis, axonal degeneration, neuroinflammation, and aberrant glial cell activation (Gunner et al., 2025). In patients with ALS, platelets are activated (Kiktenko et al., 2005; Strijkova et al., 2022). Activation of platelets leads to neuronal excitotoxicity, disruption of the vascular microenvironment, and mitochondrial metabolic failure, all of which cause damage to motor neurons and jointly accelerate the progression of ALS. First, the level of glutamine synthetase is abnormally elevated in these activated platelets, leading to glutamate excitotoxicity and accelerating excitotoxic damage to motor neurons. Second, the release of glycoproteins from platelet *α*-granules is significantly increased in ALS patients. Platelet aggregation induces vascular damage and disrupts the blood–brain barrier, further compromising the survival microenvironment of motor neurons (Espinosa-Parrilla et al., 2019; Saul et al., 2020). In addition, platelet mitochondrial dysfunction—itself closely linked to systemic metabolic derangements in ALS—adds an energetic failure component to the neurodegenerative cascade (Shrivastava et al., 2011; Ehinger et al., 2015). The important point is that these three pathways do not function independently but are interrelated. Of these, platelets occupy a crucial role, acting as both indicators of central pathology and active drivers of disease progression.

### 4.5 Platelet activation and Huntington’s disease (HD)

HD is an autosomal dominant neurodegenerative disorder caused by the aggregation of mutant huntingtin (mHtt) protein (Ananbeh et al., 2021). Platelets express the highest levels of huntingtin protein among peripheral blood cells, and mHtt localizes to the plasma membrane, *α*-granules, and open canalicular system. Upon, activation, these platelets release enhanced quantities of factors that promote mHtt aggregation or toxicity in neurons, thereby directly contributing to neuronal dysfunction and death—the hallmark of HD (Denis et al., 2019). In addition, platelets harbor tau proteins whose abnormal phosphorylation and truncation, detaches from microtubules (MTs), and forms intracellular aggregates. The resulting collapse of microtubules and disruption of axonal transport leads to loss of cell integrity and neuronal dysfunction (Alpaugh et al., 2025). These platelet-derived mHtt and tau pathologies constitute a peripheral reservoir of neurotoxic proteins that, upon activation, deliver a dual pathogenic assault on neurons, linking platelet activation to HD progression.

In conclusion, platelet activation contributes to the pathogenesis of various neurodegenerative diseases. A strong correlation exists between platelets and the nervous system (Wei and Tang, 2025). Activated platelets serve as a key driver of cognitive and motor decline through mechanisms including neuroinflammation, blood–brain barrier disruption, and excitotoxicity.

### 4.6 Platelet activation and cognitive impairment in MASLD

MASLD is a chronic, progressive metabolic disorder. As noted earlier, MASLD and cognitive dysfunction share common pathological pathways, including inflammatory response, insulin resistance, gut microbiota dysbiosis, vascular dysfunction and platelet activation. This suggests that the two diseases may share common preventive strategies. In MASLD patients, platelet activation and a hyperreactive state have been clearly demonstrated, along with cognitive dysfunction such as attention deficits. Furthermore, platelet activation has been confirmed to participate in the pathological processes of various neurodegenerative diseases. Therefore, incorporating MASLD-related cognitive impairments into the framework of “cognitive impairments in degenerative diseases” would facilitate systematic research by leveraging the known pathogenesis of neurodegenerative diseases. Evidence from the role of platelets in neurodegenerative diseases and the pathways linking MASLD to cognitive dysfunction suggests that platelet activation may act as a molecular link between hepatic metabolic disorders and brain dysfunction, thereby contributing to MASLD-induced cognitive impairment. (1) In the context of MASLD, platelets exhibit an activated or highly reactive state, characterized by a decreased platelet count and elevated activation markers such as PDW, MPV, and CD40L. (2) Platelet activation parameters, including MPV and PDW, are significantly correlated with cognitive decline (Xiong et al., 2025), whereas platelet count is negatively associated with cognitive impairment (Zhang et al., 2023). Platelets mediate the link between NAFLD and cerebrovascular/cognitive impairment through complementary evidence: Lombardi et al. established enhanced platelet activity and procoagulant imbalance as mechanistic drivers of cerebrovascular risk (Lombardi et al., 2019), while Guo et al. (2025) quantified, using structural equation modeling in a UK Biobank cohort, that platelet abnormalities statistically mediate liver disease effects on brain function—confirming this mediation in NAFLD patients. Importantly, inhibiting platelet activation has been shown to alleviate blood–brain barrier damage and improve cognitive decline (Xu et al., 2025). Based on the mechanisms underlying MASLD-induced cognitive impairment discussed above, activated platelets may act on the brain both indirectly and directly—via inflammatory responses, insulin resistance, immune dysregulation, cerebrovascular dysfunction, and the gut–liver–brain axis—thereby contributing to the progression of cognitive impairment in MASLD. These pieces of evidence collectively confirm that platelets are a key node in the liver–brain axis, suggesting that platelet-targeted interventions may alleviate cognitive complications associated with liver diseases.

In conclusion, platelet activation is not only a shared pathological mechanism in classic neurodegenerative diseases—including Alzheimer’s, Parkinson’s, multiple sclerosis, and amyotrophic lateral sclerosis—but also a likely key driver of cognitive decline in MASLD patients.

## 5 Conclusion

Cognitive dysfunction represents a significant extrahepatic complication of MASLD, for which no curative pharmacological interventions currently exist. Antiplatelet therapy has recently emerged as a promising therapeutic strategy for MASLD and its associated complications. Clinical evidence suggests that antiplatelet therapy may reduce liver volume and hepatic fat content in patients with MASLD complicated by cardiovascular disease (Shepardson et al., 2011). Clinical studies indicate that antiplatelet therapy could decrease liver volume and fat content among patients with NAFLD (now reclassified as MASLD) complicated by cardiovascular diseases. Preclinical studies have further demonstrated that aspirin can slow the progression of NAFLD and liver fibrosis, thereby mitigating the deleterious effects of lipotoxicity on cognitive function (Violi et al., 2024). Beyond its canonical role in reducing intrahepatic platelet aggregation and alleviating hepatic steatohepatitis, antiplatelet therapy has been shown to mitigate blood–brain barrier (BBB) disruption. This cerebroprotective effect is achieved through the inhibition of platelet activation, which reduces cerebral Aβ accumulation and attenuates hippocampal neuronal damage—ultimately preserving cognitive function. Based on these premises, antiplatelet therapy may ameliorate MASLD-associated cognitive impairment through the following potential mechanisms: (1) By suppressing platelet activation, antiplatelet therapy inhibits platelet transmigration across the BBB and curbs their direct pro-inflammatory effects within the CNS, thereby alleviating neuroinflammation. (2) Given that activated platelets frequently engage in crosstalk with immune cells, antiplatelet therapy reduces immune cell infiltration into the CNS. This dampens the activation of neuroinflammatory cascades and contributes to the preservation of cognitive function. (3) Indirectly, antiplatelet therapy facilitates the release of anti-inflammatory mediators from platelets—such as transforming growth factor-*β* (TGF-β)-which suppress microglial overactivation and attenuate neuronal injury (Boccatonda et al., 2024). (4) Additionally, by inhibiting the activation and proliferation of hepatic stellate cells (HSCs) and reducing hepatic fibrosis, antiplatelet therapy may confer cognitive benefits indirectly through the amelioration of underlying MASLD pathology (Thongtan et al., 2022). But the application of antiplatelet therapy in the context of cognitive impairment remains controversial—primarily due to the associated risk of bleeding (Kitt et al., 2023; Jacobs et al., 2015; Hilkens et al., 2021). Patients with chronic liver diseases, particularly those with MASLD or cirrhosis, often present with comorbidities including portal hypertension, esophagogastric varices, and coagulation disorders, all of which may further increase bleeding risk. Therefore, in clinical practice, the use of antiplatelet drugs requires careful balancing of therapeutic benefits against adverse effects to achieve an optimal equilibrium. Conventional antiplatelet drugs—including aspirin, clopidogrel, ticagrelor, dipyridamole, and tirofiban—are associated with well-recognized side effects. In managing the side effects of antiplatelet therapy, the “de-escalation” strategy is both effective in reducing bleeding risk and the most widely employed approach. This includes de-escalating potent P2Y₁₂ inhibitors to less potent agents, shortening DAPT duration, or transitioning to monotherapy after initial DAPT (Guo et al., 2025). There are also scenarios in which multiple drugs are administered in combination; however, such regimens may exacerbate adverse effects, including hepatotoxicity and systemic immune disorders (Alhawiti, 2026). Therefore, future antiplatelet therapies should prioritize more precise, patient-tailored strategies. Current antiplatelet therapy is expanding to new targets such as GPVI and PCSK9, with the aim of inhibiting thrombosis while reducing the risk of bleeding (Hay et al., 2022; Chen et al., 2026). Platelets have been shown to exert neuroprotective effects on glial cells by serving as carriers for mitochondrial delivery to the brain, raising the possibility that mitochondria themselves could serve as therapeutic targets for antiplatelet intervention (Inaba et al., 2020). This hypothesis is further supported by the identification of platelet mitochondrial PDK2/4 and PKM2 as critical metabolic checkpoints. Pharmacological modulation of these targets selectively suppresses pathological platelet activation and arterial thrombosis while preserving surface receptor function—a feature that enables patient stratification and paves the way for precision antiplatelet therapy (Yao et al., 2026). Ultimately, this approach offers a promising solution to the long-standing clinical dilemma of balancing antithrombotic efficacy with bleeding risk. Overall, antiplatelet therapy is advancing toward safer, more precise, and individualized regimens. However, this progress also underscores the pressing need for strategies that minimize bleeding risk, particularly in vulnerable populations such as those with MASLD, cirrhosis, or cognitive impairment—who face heightened susceptibility to both thrombosis and hemorrhage.

The pathogenesis of cognitive dysfunction in MASLD is complex and multifactorial. Although accumulating evidence supports a key regulatory role for platelets in both MASLD progression and cognitive function, they are unlikely to be the sole host cellular factors linked to MASLD-mediated cognitive decline. Other mechanisms discussed in this study—including immune cell infiltration, lipotoxicity, and the gut-brain axis—may also contribute to cognitive impairment. For instance, serum Klotho levels have been independently associated with cognitive performance in NAFLD patients, suggesting that multiple parallel pathways exist. A comprehensive investigation of all potential mediators, however, is beyond the scope of the present study. Nevertheless, platelets possess unique attributes—including their small size and ability to cross vascular barriers—that position them as critical circulating signal carriers, linking peripheral metabolic organs with the central nervous system. This concept is supported by the work of Inaba et al. (2020), who demonstrated that platelets can serve as vehicles to deliver mitochondria to ischemic brain regions, exerting neuroprotective effects. We therefore propose that platelets represent one of the signaling conduits between the liver and the brain. By targeting this intermediary role, our findings may offer novel therapeutic avenues for MASLD-associated cognitive impairment and provide potential predictive biomarkers for early diagnosis.
